# Cross-sectional study on exercise capacity in obese patients with severe obstructive sleep apnea syndrome

**DOI:** 10.3389/fphys.2025.1580308

**Published:** 2025-08-25

**Authors:** Shengrui Mei, Yue Zhou, Wenjun Wu, Xiaochuan Cui, Junyan Cai, Peng Yuan, Shukun Deng

**Affiliations:** ^1^ Department of Rehabilitation Medicine, The Affiliated Wuxi People’s Hospital of Nanjing Medical University, Wuxi People’s Hospital, Wuxi Medical Center, Nanjing Medical University, Wuxi, China; ^2^ Department of Endocrine, Jinshan Branch of Shanghai Sixth People’s Hospital, Shanghai, China; ^3^ Department of Sleep Center, The Affiliated Wuxi People’s Hospital of Nanjing Medical University, Wuxi People’s Hospital, Wuxi Medical Center, Nanjing Medical University, Wuxi, China; ^4^ Department of Rehabilitation Medicine, The Affiliated Hospital of Jiangnan University, Wuxi, China

**Keywords:** Obesity, obstructive sleep apnea syndrome, cardiopulmonary exercise test, exercise capacity, VO_2peak_%Pred

## Abstract

**Objective:**

To explore the exercise capacity in obese patients with severe obstructive sleep apnea syndrome (OSAS) through cardiopulmonary exercise test (CPET).

**Methods:**

In this cross-sectional study, patients with simple obesity (36 cases) and obese patients with severe OSAS (45 cases) admitted to the Department of Rehabilitation Medicine and the Department of General Practice of the Affiliated Wuxi People’s Hospital of Nanjing Medical University from September 2019 to January 2024 were collected. Additionally, we included 33 patients (BMI<28 kg/m^2^,AHI<5/hour) as a control group. All participants underwent CPET and polysomnography monitoring. The differences of polysomnography and CPET among the three groups were compared. To evaluate the correlation between AHI and the observed indexes.

**Results:**

No significant differences were observed in the proportion of men and women, age, height, exercise habit among the three groups (P > 0.05). Sleep monitoring data indicated that there were statistically significant differences among the three groups of patients in terms of the AHI, sleep efficiency, min SaO_2_, mean SaO_2_, and TS < 90%. In terms of the cardiopulmonary exercise test indexes, there were no statistically significant differences among the three groups of patients in VO_2AT_, while there were statistically significant differences in VO_2AT_%Pred and VO_2AT_/kg. Similar results were observed for maximum oxygen consumption, with no statistically significant differences in VO_2peak_ among the three groups, but statistically significant differences in VO_2peak_%Pred and VO_2peak_/kg. There were statistically significant differences among the three groups of patients in HR_max_,HR_max_%Pred, HRr, VO_2_/HR_max_, and VO_2_/HR_max_ %Pred. The correlation analysis indicated that AHI was positively correlated with TS < 90%,VO_2_/HR_max_, BMI and negatively correlated with sleep efficiency, minSO_2_, mean SO_2_,VO_2AT_%Pred, VO_2AT_/kg, VO_2peak_%Pred, VO_2peak_/kg, HR_max_, HR_max_%Pred,HRr, VO_2_/HR_max_%Pred, WR_max_%Pred, and VE_max_%Pred. Univariate and multivariate linear regression analyses showed that AHI was significantly negatively associated with multiple indicators of the cardiopulmonary exercise test (CPET). In the univariate model, for every 1-unit increase in AHI, all CPET indicators decreased significantly (p < 0.01). After adjusting for confounding factors such as gender, age, exercise habits, smoking history, hypertension and diabetes, the multivariate model still maintained significant correlations.

**Conclusion:**

Severe OSAS, as a severe complication of obesity, further exacerbates the decline in exercise capacity among obese patients, with the extent of impairment positively correlated with AHI values.CPET can be used to assess cardiopulmonary function in patients with obesity and OSAS. Early intervention can be carried out for obese patients with severe OSAS who show a downward trend in cardiopulmonary exercise indicators.

## 1 Introduction

Obesity is a chronic metabolic disease characterized by excessive accumulation of body fat to a degree that poses significant risks to health. It not only affects physical appearance but is also closely associated with various serious health problems, making it a major global public health issue (Gillison et al., 2022; Jebeile et al., 2022). Common complications of obesity include type 2 diabetes, hypertension, coronary heart disease, stroke, Obstructive Sleep Apnea Syndrome (OSAS), and others (Kim, Lim, and Kim, 2021; Bachrach et al., 2022). BMI(Body Mass Index) is used to assess the relationship between weight and height, serving as a simple tool to determine whether an individual is obese.Based on the Chinese standards (Zeng et al., 2021) and the Expert Consensus on the Prevention and Treatment of Obesity in Chinese Adults released by the Obesity Group of the Chinese Medical Association Endocrinology Branch in 2011, a BMI≥28 kg/m^2^ indicates obesity.

OSAS is a common sleep disorder characterized by repeated episodes of breathing cessation or hypopnea during sleep (Lee and Sundar, 2021). AHI (Apnea-Hypopnea Index) represents the number of apnea and hypopnea events per hour of sleep, which is a key indicator for assessing the severity of OSAS.AHI = (Number of apneas + Number of hypopneas)/Total sleep time. Based on the AHI value, the severity of OSAS is classified into the following categories:nomal (AHI <5/hour), mild OSAS (AHI 5–14.9/hour), moderate OSAS (AHI 15–29.9/hour), and severe OSAS (>30/hour) (Lee and Sundar, 2021; Challamel et al., 2021). In obese patients, the incidence of OSAS can be as high as 60%,which is significantly elevating the risk of cardiovascular diseases, metabolic dysregulation, neurological impairments, and mental health disorders, thereby profoundly compromising their quality of life (Peker et al., 2023; Dissanayake et al., 2022; Bock et al., 2021). Obesity and OSAS are mutually influencing factors.In OSAS patients, approximately 20% are obese (Antonaglia and Passuti, 2022). Our previous research has demonstrated that aerobic capacity tends to decline in obese patients (Deng et al., 2024). The impact of OSAS on exercise capacity has yielded contradictory results in current research. Some studies suggest it leads to a decline in exercise ability (Beitler et al., 2014; Van Offenwert et al., 2019; Wåhlin Larsson et al., 2008), while others indicate no effect on exercise capacity (Powell et al., 2019). Research on the exercise capacity of obese patients with OSAS is relatively scarce.

Cardiopulmonary exercise testing (CPET) is a method used to assess the heart, lung, and circulatory system’s function during progressively increasing exercise (Sherman and Saggar, 2023). By analyzing various parameters, CPET provides insights into the interplay and reserve capacity of these systems. It can yield data such as peak oxygen consumption and oxygen pulse, which are used to comprehensively evaluate a patient’s exercise capacity.

Obesity and OSAS are linked to many other chronic diseases and consume substantial medical resources, making early intervention particularly important. The purpose of this study is to understand the exercise capacity of obese patients with severe OSAS, as well as the relationship between AHI and exercise capacity, thereby guiding early interventions for obesity and OSAS patients.

## 2 Materials and methods

### 2.1 Participants and study design

This cross-sectional study retrospectively analyzed all participants who underwent both polysomnography (PSG) and cardiopulmonary exercise testing (CPET) at Wuxi People’s Hospital Affiliated to Nanjing Medical University from September 2019 to January 2024. After applying the predefined inclusion/exclusion criteria, patients with simple obesity (36 cases, BMI≥28 kg/m^2^ and AHI<5/hour) and obese patients with severe OSAS (45 cases, BMI≥28 kg/m^2^ and AHI>30/hour) were included. Additionally, we included 33 patients (BMI<28 kg/m^2^,AHI<5/hour) as a control group. This study was conducted in accordance with the Declaration of Helsinki. Protocols involving human participants were reviewed and approved by the Institutional Ethics Committee of the Affiliated Wuxi People’s Hospital of Nanjing Medical University (KYLLKS201806, KS2019020).

The inclusion criteria were as follows: 1) age between 18 and 60 years; 2) available polysomnography data; 3) available cardiopulmonary exercise test data; 4) all participants signed informed consent forms. The exclusion criteria were as follows: 1) heart, lung, or musculoskeletal diseases that would affect the conduct of the cardiopulmonary exercise test; 2) use of medications that affect heart rate, such as β receptor blockers; 3) patients with rheumatism, immunological diseases, hematological diseases, or malignant tumors; 4) secondary obesity patients, including those with hypothyroidism, Cushing’s syndrome, or long-term use of medications that cause obesity; 5) patients who are unable to tolerate or complete polysomnography monitoring; 6) Participants with RER <1.1 in the CPET test.

### 2.2 Retrospective

This study presented a retrospective analysis of data derived from subjects who fulfilled the specified inclusion and exclusion criteria and underwent cardiopulmonary exercise testing at the Rehabilitation Department, polysomnography monitoring at the sleep center of the Department of General Medicine of Wuxi People’s Hospital affiliated with Nanjing Medical University, spanning from September 2019 to January 2024.

### 2.3 Sample size calculation

In this study, to ensure statistical power and the reliability of the results, we utilized the PASS 15.0 software for sample size estimation. Based on the results of the preliminary trial, we selected the mean VO_2peak_%Pred values for three groups of participants to estimate the sample size (Vecchiato et al., 2022). We set the significance level (α) at 0.05 and the statistical power (1-β) at 0.90. The results indicated that under these conditions, at least 27 participants per group were required to achieve the desired statistical power. We then compiled the baseline data (such as gender composition, age, height, history of smoking, comorbidities and exercise habits) of a total of 114 participants. We found that the number of participants included in each of the three groups was greater than 27 (n = 33/36/45), and there were no statistically significant differences in the baseline data of these 114 participants across the three groups (P < 0.05). Therefore, a total of 114 participants were ultimately included.

### 2.4 Polysomnography

All participants underwent polysomnography monitoring (Alice 5, Philips Respironics, United States), and the data were analyzed by an experienced sleep technician (a certified healthcare professional who specializes in performing and analyzing polysomnography to diagnose sleep disorders). During polysomnography (PSG) testing, the environment must strictly simulate ideal sleep conditions to ensure accurate results: room temperature maintained at 20 °C–24 °C, humidity at 40%–60%, complete darkness, private room with comfortable bedding, and minimized external disturbances. The data were recorded for AHI, sleep efficiency (total sleep time/total time in bed), min SaO_2_ (minimum oxygen saturation), mean SaO_2_ (average oxygen saturation), TS < 90% (the percentage of time with oxygen saturation below 90%). Apneas were defined as a complete cessation of airflow for at least 10 s. Hypopneas were defined as a reduction in airflow of ≥30%, lasting for at least 10 s, accompanied by a drop in oxygen saturation of ≥3%. The AHI (Apnea-Hypopnea Index), which is the average number of apneas and hypopneas per hour of sleep, served as the primary indicator for diagnosing OSAS. The diagnostic criterion for OSAS is an AHI of ≥5/hour.

### 2.5 The cardiopulmonary exercise test

All participants were evaluated with cardiopulmonary exercise tests. The test was performed by the CS-200 exercise cardiopulmonary tester (Schiller, Switzerland). The participants underwent a symptom-limited cardiopulmonary exercise test.A trained professional rehabilitation physician performs a physical examination, history, and cardiopulmonary exercise test measurements. Prior to testing, the equipment underwent standardized preparation involving a 15-min preheating phase followed by sequential calibration procedures: temperature calibration (ambient range 20 °C–25 °C), humidity adjustment (40%–60% RH), volumetric calibration using a 3L syringe, and metabolic preconditioning verification. Participants initially completed resting-state assessments comprising 12-lead electrocardiogram (ECG) monitoring and baseline pulmonary function tests. The workload selection for cardiopulmonary exercise testing (CPET) is determined based on the patient’s anthropometric parameters (height, weight, age,gender) to derive their predicted peak power output. This reference value is divided by 10 to establish the wattage ramp increment protocol. Typically, a 10–15 W/min gradient is applied. Then, the cycling test begined. During the cycling test, the participant starts with a 3-min warm-up (0W), after which the exercise intensity was gradually increased (10–15 W/min increment) until the participant reaches their limit (when participants reached volitional exhaustion, experienced adverse symptoms such as palpitations, dizziness, or encountered any condition precluding further exercise). After the exercise, a 3-min recovery period was conducted. Throughout this process, the participant’s heart rate, electrocardiogram (ECG), oxygen conconsumption (VO_2_), carbon dioxide output (VCO_2_), and other indicators were monitored.

The cardiopulmonary exercise test-related indicators were collected, including VO_2_ at anaerobic threshold (VO_2AT_), percentage of predicted VO_2AT_ (VO_2AT_% Pred), peak oxygen consumption (VO_2peak_), percentage of predicted VO_2peak_ (VO_2peak_% Pred), peak kilogram oxygen consumption (VO_2peak_/kg), maximum exercise power (WR_max_), breathing reserve (BR), maximum heart rate (HR_max_), percentage of predicted HRmax (HR_max_% Pred), maximum O_2_ pulse (VO_2_/HR_max_), percentage of predicted maximum O_2_ pulse (VO_2_/HR_max_% Pred), Heart rate response (HRr)= (HR_max_-HR_rest_)/(VO_2peak_-VO_2rest_),forced vital capacity (FVC), ratio of forced expiratory volume to vital capacity in 1 s (FEV1/FVC), percentage of predicted forced vital capacity (FVC% Pred), percentage of predicted forced expiratory volume ratio of 1 s (FEV1% Pred), and peak expiratory flow rate (PEF), maximum ventilatory volume (MVV).During the exercise, the following parameters were also measured: maximum exercise ventilation (VE_max_), the percentage of maximum exercise ventilation relative to the predicted value (VE_max_%Pred) and ventilation efficiency (VE/VCO_2_ slope).

### 2.6 Statistical analysis

Continuous variables were assessed for normality using the Shapiro-Wilk test. Data that conformed to a normal distribution were expressed as means and standard deviations, and group comparisons were made using one-way ANOVA, with pairwise comparisons conducted using the Tambane’s T2 method. For data that did not meet the normality assumption, the Kruskal–Wallis test was used for group comparisons, and the median (P25, P75) was used to represent the data. Categorical data were expressed as rates and compared between groups using the chi-square test. Spearman correlation analysis was used to analyze the correlation between AHI and each indexes. P < 0.05 was considered statistically significant. Then, univariate regression analysis was further conducted on the factors with p < 0.001. To eliminate the influence of confounding factors, multivariate regression analysis was performed on AHI, history of smoking, gender, age, exercise habits, history of hypertension, and history of diabetes to determine the relationship between the severity of AHI and various factors of CPET. To better approximate the normal distribution, logarithmic transformation was performed on AHI in all regression analyses. All statistical analyses were conducted using SPSS 25.0.

## 3 Result

### 3.1 Comparison of general information for groups

General demographic data were collected for all participants, including age, gender, height, weight, BMI, and exercise habits. We used the International Physical Activity Questionnaire (IPAQ-SF) to assess exercise habits, and individuals who reached a moderate activity level (≥600 MET-min/week) or higher were considered to have an exercise habit (Roberts-Lewis et al., 2022). The control group, OB group and OB-severe OSAS group comprised 26 men and 7 women,28 men and 8 women, 40 men and 5 women, respectively (P = 0.343). The age in the control group, OB group and OB-severe OSAS group were 36.64 ± 7.90, 35.72 ± 8.28, 38.93 ± 9.17, respectively (P = 0.220). No significant differences were observed in the proportion of men and women, age, height, exercise habit among the three groups (P > 0.05). The weight in the control group, OB group and OB-severe OSAS group were 69.67 ± 12.06, 104.07 ± 20.41, 97.49 ± 16.13, the differences were statistically significant (P < 0.001), but there is no significant difference between the OB group and the OB-severe OSAS group (P = 0.080). The BMI in the control group, OB group and OB-severe OSAS group were 24.60 (21.15, 26.35), 35.45 (32.43, 40.08), 31.70 (30.00, 35.05), the differences were statistically significant (P < 0.001), but there is no significant difference between the OB group and the OB-severe OSAS group (P = 0.075). There were no significant differences among the three groups in the prevalence of hypertension (control group: 39.4%, OB group: 47.2%, OB-severe OSAS group: 46.7%; χ^2^ = 0.539, P = 0.764), diabetes (control group: 30.3%, OB group: 38.9%, OB-severe OSAS group: 46.7%; χ^2^ = 2.141, P = 0.343), or history of smoking (control group: 36.4%, OB group: 33.3%, OB-severe OSAS group: 51.1%; χ^2^ = 3.062, P = 0.216) (Table 1).

**TABLE 1:** Comparison of general information for groups.

Items	control (n = 33)	OB(n = 36)	OB-severe OSAS(n = 45)	χ2 or F or H	P
Age (years old)	36.64 ± 7.90	35.72 ± 8.28	38.93 ± 9.17	F = 1.536	0.220
Gender, male (%)	26 (78.8%)	28 (77.8%)	40 (88.9%%)	χ2 = 2.139	0.343
Height (cm)	169.67 ± 6.96	168.69 ± 8.44	172.29 ± 6.87	F = 2.567	0.081
Weight (kg)	69.67 ± 12.06	104.07 ± 20.41	97.49 ± 16.13	F = 41.661	<0.001
BMI(kg/m^2^)	24.60 (21.15,26.35)	35.45 (32.43,40.08)	31.70 (30.00,35.05)	H = 74.451	<0.001
Exercise habit,yes (%)	17 (51.5%)	17 (47.2%)	33 (73.3%)	χ2 = 5.916	0.052
Hypertension,yes (%)	13 (39.4%)	17 (47.2%)	21 (46.7%)	χ2 = 0.539	0.764
Diabetes,yes (%)	10 (30.3%)	14 (38.9%)	21 (46.7%)	χ2 = 2.141	0.343
History of smoking,yes (%)	12 (36.4%)	12 (33.3%)	23 (51.1%)	χ2 = 3.062	0.216

### 3.2 Comparison of sleep monitoring for groups

Sleep monitoring data indicated that there were statistically significant differences among the three groups of patients in terms of the AHI, sleep efficiency, min SaO2, mean SaO2, and TS < 90%. There were no significant differences in these indicators between the control group and the OB group, while the OB-severe OSAS group showed statistically significant differences in these indicators compared to both the control group and the OB group (Table 2).

**TABLE 2:** Comparison of sleep monitoring for groups.

Items	control (n = 33)	OB(n = 36)	OB-severe OSAS(n = 45)	H	P
AHI(events/h)	0 (0,2)	0 (2,2.68)	66.70 (47.30,82.00)	H = 86.352	<0.001^b^ ^,^ ^c^
Sleep efficiency (%)	97.66 (96.12,98.27)	98.00 (97.48,98.82)	93.44 (87.52,96.39)	H = 32.275	<0.001^b^ ^,^ ^c^
minSaO_2_ (%)	90.00 (87.00,92.00)	92.00 (87.00,93.00)	61.00 (54.00,72.50)	H = 77.950	<0.001^b^ ^,^ ^c^
meanSaO_2_ (%)	93.00 (92.00,94.50)	94.00 (93.00,95.00)	90.50 (87.00,92.00)	H = 41.842	<0.001^b^ ^,^ ^c^
TS < 90% (%)	0.63 (0.09,1.47)	0.2 (0,1.17)	11.97 (0.51,36.60)	H = 23.066	<0.001^b^ ^,^ ^c^

Note.

^a^ P < 0.05 between control and OB.

^b^ P < 0.05 between control and OB-OSAS.

^c^ P< 0.05 between OB, and OB-severe OSAS.

### 3.3 Comparison of cardiopulmonary exercise test indexes

**FIGURE 1:**
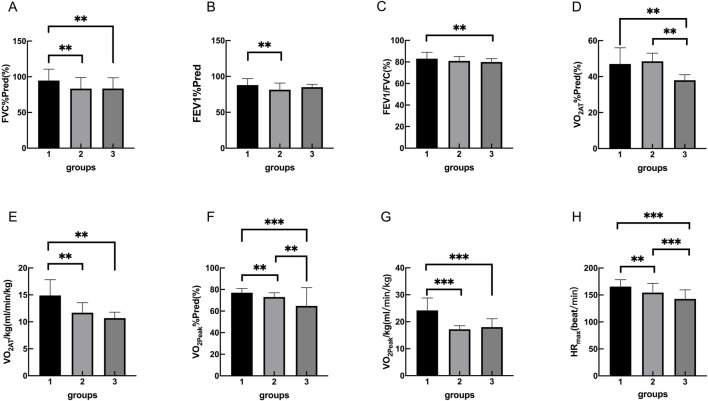
Group 1, 2, 3 represents the control group, OB group, OB-severe OSAS group.respectively. The comparison of cardiopulmonary exercise test indexes for three groups on FVC%Pred **(A)**, FEV1%Pred **(B)**, FEV1/FVC **(C)**, VO_2AT_%Pred **(D)**, VO_2AT_/kg **(E)**, VO_2peak_%Pred **(F)**, VO_2peak_/kg **(G)**, HR_max_ **(H)**. **p < 0.05, ***p < 0.001.

The static pulmonary function showed that the FVC%Pred, FEV1%Pred and FEV1/FVC were significantly different among the three groups (P < 0.05). Moreover, there were significantly different in FVC% Pred between control group and OB group, control group and OB-severe OSAS group (P < 0.05), and there was no significantly different between OB group and OB-severe OSAS group (P = 0.968). There were significantly different in FEV1% Pred between control group and OB group (P < 0.05), and there was no significantly different between control group and OB-severe OSAS group (P = 0.207), OB group and OB-severe OSAS group (P = 1.000). There were significantly different in FEV1/FVC between control group and OB-severe OSAS group (P < 0.05), and there was no significantly different between control group and OB group (P = 0.073),OB group and OB- severe OSAS group (P = 1.000) (Table 3; Table 4; Figure 1).

**TABLE 3:** Comparison of cardiopulmonary exercise test indexes.

Items	control (n = 33)	OB(n = 36)	OB- severe OSAS(n = 45)	F or H	P
FVC(L)	3.59 (3.13,4.31)	3.26 (2.65,3.89)	3.73 (3.10,4.47)	H = 5.140	0.077
FVC%Pred (%)	94.70 ± 16.00	83.42 ± 15.47	83.56 ± 15.11	F = 6.139	0.003^a^ ^,^ ^b^
FEV1%Pred (%)	88.00 (83.00,99.00)	81.50 (70.50,90.75)	85.00 (75.00,91.50)	H = 6.363	0.042^a^
FEV1/FVC(%)	83.00 (79.00,90.50)	81.00 (75.00,85.00)	80.00 (74.50,87.00)	H = 6.167	0.046
PEF (L/s)	6.04 ± 2.02	5.15 ± 1.79	6.05 ± 2.14	F = 2.473	0.089
PEF%Pred (%)	76.00 (61.00,87.00)	68.50 (50.00,77.75)	74.00 (61.00,83.50)	H = 4.215	0.121
MVV(L/min)	100.45 (89.25,127.40)	98.88 (78.40,112.00)	109.90 (91.53,128.28)	H = 4.293	0.117
VO_2AT_(L/min)	1.08 (0.83,1.28)	1.17 (0.96,1.24)	1.04 (0.88,1.25)	H = 2.024	0.364
VO_2AT_%Pred (%)	47.00 (42.00,56.00)	48.50 (41.00,56.75)	38.00 (32.00,44.00)	H = 20.035	<0.001^b^ ^,^ ^c^
VO_2AT_/kg (mL/min/kg)	14.90 (13.05,18.20)	11.70 (10.08,13.55)	10.70 (9.15,12.25)	H = 36.016	<0.001^a^ ^,^ ^b^
VO_2peak_ (L/min)	1.80 ± 0.42	1.77 ± 0.41	1.77 ± 0.45	F = 0.050	0.951
VO_2peak_%Pred (%)	78.94 ± 10.78	71.86 ± 11.64	64.80 ± 16.88	F = 10.137	<0.001^a^ ^,^ ^b^ ^,^ ^c^
VO_2peak_/kg (mL/min/kg)	24.20 (21.20,29.95)	17.20 (15.70,20.05)	18.00 (15.25,21.10)	H = 43.168	<0.001^a^ ^,^ ^b^
HR_max_ (beat/min)	165.55 ± 12.68	154.31 ± 17.20	142.73 ± 16.69	F = 19.991	<0.001^a^ ^,^ ^b^ ^,^ ^c^
HR_max_%Pred (%)	91.24 ± 9.00	81.53 ± 9.69	79.56 ± 7.74	F = 18.369	<0.001^a^ ^,^ ^b^
RER_max_	1.28 (1.18,1.35)	1.25 (1.20,1.33)	1.27 (1.23,1.42)	H = 3.106	0.212
HRr	55.97 (44.27,67.33)	45.48 (30.06,66.40)	41.56 (29.29,62.12)	H = 9.805	0.007^b^
VO_2_/HR_max_ (ml/beat)	9.60 (8.30,11.85)	10.40 (9.43,14.05)	12.30 (10.10,14.45)	H = 9.971	0.007^b^
VO_2_/HR_max_%Pred (%)	79.00 (68.50,89.50)	65.00 (53.25,77.75)	59.00 (48.50,69.50)	H = 20.188	<0.001^a^ ^,^ ^b^
BR (%)	50.79 ± 14.91	43.61 ± 16.72	47.60 ± 17.91	F = 1.615	0.204
WR_max_ (W)	126.00 (113.5,159.00)	125.00 (112.75,152.00)	146.00 (117.00,175.00)	H = 2.252	0.324
WR_max_%Pred (%)	84.03 ± 13.24	71.47 ± 11.47	66.96 ± 12.91	F = 18.130	<0.001^a^ ^,^ ^b^
VE_max_ (L/min)	51.97 (42.34,64.00)	51.98 (42.97,65.39)	55.29 (47.24,71.69)	H = 3.245	0.197
VE_max_%Pred (%)	76.39 ± 14.14	57.97 ± 17.47	55.38 ± 16.20	F = 18.196	<0.001^a^ ^,^ ^b^
VE/VCO_2_ slope	22.30 (18.55,22.83)	22.02 (19.78,24.00)	23.07 (20.92,26.83)	H = 5.067	0.079

Note.

^a^ P < 0.05 between control and OB.

^b^ P < 0.05 between control and OB- severe OSAS.

^c^ P< 0.05 between OB, and OB-severe OSAS.

**TABLE 4:** Intergroup comparison of cardiopulmonary exercise test indexes.

Intergroup comparison		FVC%Pred (%)		FEV1%Pred (%)		FEV1/FVC(%)		VO_2AT_%Pred (%)	
		Mean difference	P	H	P	H	P	H	P
control	OB	11.280	0.003	19.580	0.042	16.794	0.104	1.525	1.000
	OB- severe OSAS	11.141	0.002	13.777	0.207	17.041	0.073	29.103	<0.001
OB	OB- severe OSAS	−0.138	0.968	−5.803	1.000	0.247	1.000	27.578	0.001

Intergroup comparison		VO_2AT_/kg (mL/min/kg)		VO_2peak_%Pred (%)		VO_2peak_/kg (mL/min/kg)		HR_max_ (beat/min)	
		H	P	Mean difference	P	H	P	Mean difference	P
control	OB	33.914	<0.001	7.078	0.035	46.207	<0.001	11.240	0.004
	OB- severe OSAS	43.814	<0.001	14.139	<0.001	43.640	<0.001	22.812	<0.001
OB	OB- severe OSAS	7.900	0.855	−7.061	0.024	−2.567	1.000	11.572	0.001

Intergroup comparison		HR_max_%Pred (%)		HRr		VO_2_/HR_max_ (ml/beat)		VO_2_/HR_max_%Pred (%)	
		Mean difference	P	H	P	H	P	H	P
control	OB	9.715	<0.001	16.711	0.108	−12.975	0.310	29.758	0.001
	OB- severe OSAS	11.687	<0.001	23.441	0.006	−23.897	0.005	41.024	<0.001
OB	OB- severe OSAS	1.972	0.316	6.731	1.000	−10.922	0.418	11.267	0.382

Intergroup comparison		WR_max_%Pred (%)		VE_max_%Pred (%)	
		Mean difference	P	Mean difference	P
control	OB	12.558	<0.001	18.422	<0.001
	OB- severe OSAS	17.075	<0.001	21.016	<0.001
OB	OB- severe OSAS	4.517	0.111	2.594	0.472

**FIGURE 2:**
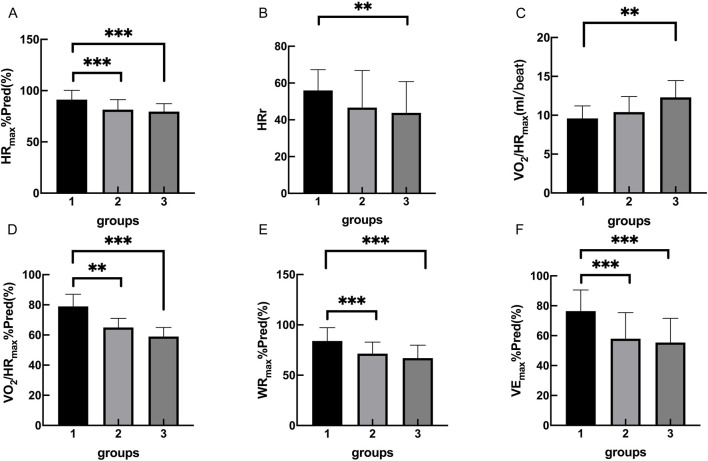
Group 1, 2, 3 represents the control group, OB group, OB-severe OSAS group.respectively. The comparison of cardiopulmonary exercise test indexes for five groups on HR_max_%Pred **(A)**, HRr **(B)**, VO_2_/HR_max_ **(C)**, VO_2_/HR_max_%Pred **(D)**, WR_max_%Pred **(E)** VE_max_%Pred **(F)**. **p < 0.05, ***p < 0.001.

The cardiopulmonary exercise test results indicated that in terms of the anaerobic threshold, there were no statistically significant differences among the three groups of patients in VO_2AT_, while there were statistically significant differences in VO_2AT_%Pred and VO_2AT_/kg. Similar results were observed for maximum oxygen consumption, with no statistically significant differences in VO_2peak_ among the three groups, but statistically significant differences in VO_2peak_%Pred and VO_2peak_/kg. In terms of cardiac function, there were statistically significant differences among the three groups of patients in HR_max_, HR_max_%Pred, HRr, VO_2_/HR_max_, and VO_2_/HR_max_ %Pred. In terms of ventilation efficiency,there were statistically significant differences among the three groups of patients in VE_max_%Pred, while there were no statistically significant differences among the three groups of patients in VE_max_ and VE/VCO_2_ slope (Tables 3, 4; Figures 1, 2).

### 3.4 Correlation analysis between AHI and the observed indexes

AHI was positively correlated with TS < 90%,VO_2_/HR_max_, BMI and negatively correlated with sleep efficiency, minSO_2_, mean SO_2_,VO_2AT_%Pred, VO_2AT_/kg, VO_2peak_%Pred, VO_2peak_/kg, HR_max_, HR_max_%Pred,HRr, VO_2_/HR_max_%Pred, WR_max_%Pred, and VE_max_%Pred (Table 5).

**TABLE 5:** Correlation analysis between AHI and the observed indexes.

Items	AHI	
	Correlation coefficient (r)	p values
Sleep efficiency (%)	−0.507**	<0.001
minSaO_2_ (%)	−0.737**	<0.001
meanSaO_2_ (%)	−0.651**	<0.001
TS < 90% (%)	0.422**	<0.001
VO_2AT_(L/min)	−0.002	0.979
VO_2AT_%Pred (%)	−0.351**	<0.001
VO_2AT_/kg (mL/min/kg)	−0.322**	<0.001
VO_2peak_ (L/min)	−0.002	0.985
VO_2peak_%Pred (%)	−0.349**	<0.001
VO_2peak_/kg (mL/min/kg)	−0.273**	0.003
HR_max_ (beat/min)	−0.434**	<0.001
HR_max_%Pred (%)	−0.340**	<0.001
RER_max_	−0.110	0.243
HRr	−0.200*	0.033
VO_2_/HR_max_ (ml/beat)	0.247**	0.008
VO_2_/HR_max_ Pred (%)	−0.354**	<0.001
BR (%)	0.090	0.340
WR_max_ (W)	0.110	0.245
WR_max_%Pred (%)	−0.377**	<0.001
VE_max_ (L/min)	−0.077	0.416
VE_max_%Pred (%)	−0.359**	<0.001
VE/VCO2 slope	−0.104	0.273
FVC(L)	0.164	0.080
FVC%Pred (%)	−0.128	0.175
FEV1%Pred (%)	−0.035	0.709
FEV1/FVC(%)	−0.130	0.169
PEF (L/s)	0.103	0.274
PEF%Pred (%)	0.010	0.915
BMI(kg/m^2^)	0.240*	0.010

Note: **p < 0.01,*p < 0.05.

### 3.5 Univariate and multivariate linear regression analyses of AHI association with CPET parameters

Univariate and multivariate linear regression analyses showed that AHI was significantly negatively associated with multiple indicators of the cardiopulmonary exercise test (CPET). In the univariate model, for every 1-unit increase in AHI, all CPET indicators decreased significantly (p < 0.01). After adjusting for confounding factors such as gender, age, exercise habits, smoking history, hypertension and diabetes, the multivariate model still maintained significant correlations. Specifically, AHI and VO_2AT_%Pred (B = −5.292, P = 0.001, adj.*R* ^2^ = 0.143), VO_2AT_/kg (B = −1.661, P = 0.002, adj.*R* ^2^ = 0.074), VO_2peak_%Pred (B = −5.956) P = 0.001, adj.*R* ^2^ = 0.133), HRmax (B = −8.481, P < 0.001, adj.*R* ^2^ = 0.245), HR_max_%Pred (B = −3.950, P = 0.001, adj.*R* ^2^ = 0.096), VO_2_/HR_max_Pred (B = -6.748, P = 0.002, adj.*R* ^2^ = 0.117), WRmax%Pred (B = −6.121, P < 0.001, adj.*R* ^2^ = 0.191) and VE_max_%Pred (B = −7.307, P = 0.001, adj.*R* ^2^ = 0.123) were negatively correlated (Table 6).

**TABLE 6:** Univariate and multivariate linear regression analyses of AHI association with CPET parameters.

Items	Univariate model					Multivariate model				
	B	95%CI	*R* ^2^	t	p	B	95%CI	Adj.*R* ^2^	t	p
VO_2AT_%Pred (%)	−5.301	(-8.146,-2.457)	0.109	−3.693	<0.001	−5.292	(-8,216,-2.367)	0.143	−3.587	0.001
VO_2AT_/kg (mL/min/kg)	−1.681	(-2.678,-0.685)	0.091	−3.343	0.001	−1.661	(-2.715,-0.606)	0.074	−3.122	0.002
VO_2peak_%Pred (%)	−6.063	(-9.264, −2.861)	0.112	−3.752	<0.001	−5.956	(-9.272, −2.639)	0.133	−3.560	0.001
HR_max_ (beat/min)	−9.970	(-13.720, −6.221)	0.199	−5.269	<0.001	−8.481	(-12.297, −4.664)	0.245	−4.406	<0.001
HR_max_%Pred (%)	−4.209	(-6.364, −2.055)	0.118	−3.870	<0.001	−3.950	(-6.237, −1.662)	0.096	−3.423	0.001
VO_2_/HR_max_Pred (%)	−6.898	(-10.878, −2.917)	0.095	−3.433	0.001	−6.748	(-10.873, −2.623)	0.117	−3.423	0.002
WR_max_%Pred (%)	−6.742	(-9.778, −3.705)	0.147	−4.399	<0.001	−6.121	(-9.222, −3.020)	0.191	−3.914	<0.001
VE_max_%Pred (%)	−7.793	(-11.734, −3.852)	0.121	−3.918	<0.001	−7.307	(-11.435, −3.179)	0.123	−3.510	0.001

## 4 Discussion

OSAS is indeed recognized as a disease that can affect multiple systems within the body, including respiratory system, cardiovascular system and metabolic system (Khurana et al., 2021). OSAS is closely linked to obesity. The obesity is a risk factor for OSAS, and in obese patients, the incidence of OSAS can be as high as 60%. Its prevalence is increasing in tandem with the severity of obesity (Jehan et al., 2017). Our correlation study findings indicate a positive correlation between BMI and AHI, highlighting the significant role of obesity in the pathogenesis of OSAS. The possible reasons are as follows: ①Fat Accumulation: Obese individuals tend to have higher fat accumulation, particularly abdominal fat deposition and visceral fat (Zhao et al., 2019). A cross-sectional study by Bingwei Ma et al. indicates that obese patients have a higher prevalence of OSAS, which is associated with abdominal fat deposition, particularly visceral fat (Ma et al., 2022). On the other hand, fat accumulation around the neck and upper airway can compress the airway, leading to narrowing or obstruction. ②Weight Gain:Weight gain often accompanies an increase in neck circumference, which further increases the risk of airway compression. ③Inflammatory Response:Obese individuals often have chronic low-grade inflammation, which can affect the structure and function of the airway, thereby increasing the incidence of OSAS (Andersen, Murphy, and Fernandez, 2016).

Obesity was associated with reduced exercise capacity, as evidenced by lower VO_2AT_%Pred, VO_2AT_/kg, VO_2peak_%Pred, and VO_2peak_/kg in the OB and OB-severe OSAS groups compared to the control group. These findings are consistent with our previous studies showing that excess body weight imposes a mechanical load on the cardiopulmonary system, leading to decreased aerobic capacity and endurance (Deng et al., 2024). Notably, the absence of significant differences in static pulmonary function (FVC%Pred, FEV1%Pred, and FEV1/FVC) between the OB and OB-severe OSAS groups suggests that obesity itself, rather than OSAS severity, is the primary contributor to impaired pulmonary mechanics. However, the reduced ventilatory efficiency (VE_max_%Pred) observed in obese individuals indicates that obesity may also impair the ability to meet increased ventilatory demands during exercise. Heart rate response (HRr) reflects the dynamic alterations in cardiac rhythm induced by physiological or psychological stressors, serving as a critical determinant of exercise tolerance (Ishihara et al., 2019). In this study, the HRr of OB group decreased compared with the control group, indicating that obese patients had lower exercise endurance. Oxygen pulse reflects the levels of cardiac output and cardiac reserve capacity, and serves as a key indicator of cardiorespiratory function under maximal load (Huang et al., 2025). The VO_2_/HR_max_%Pred of the OB group was lower than that of the control group, indicating that obesity would affect the cardiopulmonary function of the patients.

In this study, compared to the OB group, the OB-severe OSAS group showed reductions in VO_2peak_%Pred, VO_2AT_%Pred. This indicated that severe OSAS further exacerbates the decline in exercise capacity among obese patients. VO_2peak_ is the peak oxygen consumption that an individual can achieve at peak exercise intensity (Simmons et al., 2000). VO_2peak_%Pred refers to the percentage of predicted peak oxygen consumption based on individual characteristics such as age, gender, and body weight.Compared to VO_2peak_,VO_2peak_%Pred is more convenient for comparing the differences in exercise capacity among different individuals, as it can eliminate the influence of individual differences. VO_2peak_%Pred can reflect the maximum aerobic exercise capacity of an individual and is an important index to evaluate cardiopulmonary endurance (Stubbe et al., 2022; Chiaranda et al., 2020). VO_2AT_ serves as a crucial indicator of aerobic capacity, reflecting the muscle mitochondria’s ability to utilize oxygen (Masuda et al., 2022). VO_2AT_ %Pred reflects differences in relative percentages due to variations in predicted values across individuals (Salvioni et al., 2022). VO_2AT_ %Pred and VO_2peak_%Pred can comprehensively reflect an individual’s aerobic exercise capacity.

The negative correlations between AHI and key cardiopulmonary exercise parameters, including VO_2AT_%Pred, VO_2peak_%Pred, HR_max_, and HR_max_%Pred, WR_max_%Pred, VO_2_/HR_max_%Pred. Univariate and multivariate regression analyses indicated that an increase in AHI independently and significantly predicted impaired cardiopulmonary exercise performance. This is manifested as a decrease in oxygen uptake, a reduction in maximum heart rate, a decrease in oxygen delivery efficiency, a decrease in maximum exercise power, and a decrease in maximum ventilation. These findings suggest that the severity of sleep breathing disorders is closely related to the reduction of cardiopulmonary reserve and exercise tolerance.

The AHI is significantly negatively correlated with key indicators of CPET, and this correlation may be jointly mediated by pathophysiological changes in multiple systems. From a cardiovascular perspective, intermittent hypoxia and sympathetic overactivation associated with OSAS can lead to vascular endothelial dysfunction and left ventricular diastolic impairment, limiting cardiac output reserve (Arnaud et al., 2020), this demonstrated a significant negative correlation between AHI and HR_max_%Pred, HR_max,_ VO_2_/HR_max_%Pred. At the skeletal muscle level, mitochondrial dysfunction and type II fiber atrophy reduce peripheral oxygen extraction efficiency, thereby resulting in decreased VO_2_AT%Pred and VO_2_peak%Pred. Additionally, ventilation-perfusion mismatch due to pulmonary vasoconstriction and heart rate response abnormalities caused by autonomic dysregulation may collectively impair oxygen delivery and utilization efficiency (Torres et al., 2024). These findings provide important pathophysiological explanations for the exercise intolerance in OSAS patients and highlight the need for clinical attention to the multisystemic impacts of OSAS. The study revealed a significant negative correlation between AHI and WR_max_%Pred, which may be attributed to multiple mechanisms: First, chronic intermittent hypoxia induces muscle atrophy and neuromuscular dysfunction (O'Halloran and Lewis, 2017). Second, metabolic disturbances in OSAS impair ATP production; third, compromised cardiopulmonary function leads to insufficient oxygen delivery; finally, increased respiratory work and reduced movement efficiency further diminish exercise performance. Collectively, these pathophysiological changes necessitate greater exertional effort in OSAS patients, resulting in markedly decreased WR_max_%Pred.

OSAS affects exercise capacity through multiple potential mechanisms, involving sleep quality, the cardiovascular system, metabolic function, neuromuscular control, and other aspects. Below are some possible mechanisms: ①Decline in Sleep Quality: OSAS patients experience frequent breathing pauses and hypopnea events, leading to poor sleep quality, frequent awakenings, and light sleep states. Prolonged sleep deprivation affects physical recovery and energy metabolism, reducing exercise endurance and performance (Rault et al., 2024). ②Damage to the Cardiovascular System: Chronic hypoxia and fluctuations in intrathoracic pressure may lead to reduced cardiac function, affecting blood circulation and oxygen delivery during exercise (Chen et al., 2020). ③ Abnormal Metabolic Function: OSAS patients may have abnormal fat metabolism, leading to weight gain and obesity, which further limits exercise capacity. Prolonged hypoxia may damage mitochondrial function, impacting aerobic metabolic capacity. ④Impaired Neuromuscular Control:Frequent breathing pauses result in hypoxemia, potentially harming brain and nervous system function, affecting exercise coordination and responsiveness. Chronic hypoxia may lead to reduced muscle function, increasing fatigue during exercise. Besides, OSAS patients often exhibit heightened sympathetic activity, which may reduce heart rate variability and affect cardiovascular regulation during exercise (Mukandala et al., 2016). ⑤Inflammation and Oxidative Stress: OSAS patients commonly experience systemic inflammatory responses, which may damage vascular endothelial function and affect blood flow during exercise (Iturriaga, Moya, and Del Rio, 2015). The mechanisms by which OSAS affects exercise capacity are multifaceted, involving sleep quality, the cardiovascular system, metabolic function, neuromuscular control, inflammation, and other factors. These elements collectively contribute to reduced endurance, increased fatigue, and significantly limited exercise capacity in patients.

CPET results can provide critical guidance for personalized interventions in obese patients with OSAS. Key parameters and their clinical implications include: VO_2peak_/kg < 17 mL/min/kg identifies individuals with significant cardiopulmonary impairment who would benefit from structured prehabilitation programs incorporating aerobic conditioning and respiratory muscle training (Guazzi et al., 2008). VO_2_AT <40%Pred reflects impaired mitochondrial oxidative capacity and warrants targeted metabolic interventions to improve anaerobic threshold (Balady et al., 2010). Heart rate recovery (1-min) <12 beats indicates sympathetic overactivation and elevated cardiovascular risk, prompting autonomic nervous system modulation strategies (Leclerc, 2017).

## 5 Conclusion

In summary, Obese patients exhibit reduced aerobic capacity compared to healthy individuals. Severe OSAS, as a severe complication of obesity, may further exacerbates the decline in exercise capacity among obese patients, with the extent of impairment positively correlated with AHI values.CPET can be used to assess cardiopulmonary function in patients with obesity and OSAS. Early intervention can be carried out for obese patients with severe OSAS who show a downward trend in cardiopulmonary exercise indicators.

## 6 Study limitation

Several limitations of this study warrant consideration. First, the study’s single-center design and relatively small cohort size may have introduced selection bias, limiting the generalizability of the findings. In future studies, we could opt for multi-center sampling. Second, while VO_2max_ is widely regarded as the gold standard for assessing maximal aerobic capacity, not all participants achieved this threshold due to technical constraints in our experimental setup. Consequently, the reliance on VO_2peak_ as a surrogate metric may underestimate true maximal exercise performance. Addressing these methodological challenges in subsequent studies would enhance the robustness of the results. In addition, when conducting the polysomnography test, we did not take into account the control of patients’ stress levels, which might affect the results of the polysomnography. We will consider this point in future research to make the data more accurate.

## Data Availability

The original contributions presented in the study are included in the article/Supplementary Material, further inquiries can be directed to the corresponding authors.
