# Supplementary material for: Cross-sectional study on exercise capacity in obese patients with severe obstructive sleep apnea syndrome
Source: Front Physiol. 2025 Aug 25;16:1580308. doi: 10.3389/fphys.2025.1580308 (PMC12415024; doi:10.3389/fphys.2025.1580308)
Supplement: Supplementary file 1 [file DataSheet1.pdf]

Supplementary Material

1.Figures and tables(This article has 6 tables and 2 figures)

Supplementary Figure 1. The figure legends are required to have the same font as the main text, 12 point normal Times New Roman, single spaced. Please use a single paragraph for each legend and prepare the figures keeping in mind the PDF layout.

Table1. Comparison of general information for groups

Items	control(n=33)	OB(n=36)	OB-severe OSAS(n=45)	χ^2 or F or H	P
Age(years old)	36.64±7.90	35.72±8.28	38.93±9.17	F=1.536	0.220
Gender,male(%)	26(78.8%)	28(77.8%)	40(88.9%%)	χ^2=2.139	0.343
Height(cm)	169.67±6.96	168.69±8.44	172.29±6.87	F=2.567	0.081
Weight(kg)	69.67±12.06	104.07±20.41	97.49±16.13	F=41.661	＜0.001
BMI(kg/m^2)	24.60(21.15,26.35)	35.45(32.43,40.08)	31.70(30.00,35.05)	H=74.451	＜0.001
Exercise habit,yes(%)	17(51.5%)	17(47.2%)	33(73.3%)	χ^2=5.916	0.052
Hypertension,yes(%)	13(39.4%)	17(47.2%)	21(46.7%)	χ^2=0.539	0.764

Diabetes,yes(%)	10(30.3%)	14(38.9%)	21(46.7%)	χ^2=2.141	0..343
History of smoking, yes(%)	12(36.4%)	12(33.3%)	23(51.1%)	χ^2=3.062	0.216

Note: ANOVA for F values; Kruskal–Wallis for H values ; Chi-square test for χ^2 values

Table 2. Comparison of sleep monitoring for groups

Items	control(n=33)	OB(n=36)	OB-severe OSAS(n=45)	H	P
AHI(events/h)	0 （0,2）	0 （2,2.68）	66.70(47.30,82.00)	H=86.352	< 0.001[bc]
Sleep efficiency (%)	97.66 （96.12,98.27）	98.00(97.48,98.82)	93.44(87.52,96.39)	H=32.275	< 0.001[bc]
minSaO2 (%)	90.00 （87.00,92.00）	92.00 （87.00,93.00）	61.00(54.00,72.50)	H=77.950	< 0.001[bc]
meanSaO2 (%)	93.00 （92.00,94.50）	94.00 （93.00,95.00）	90.50(87.00,92.00)	H=41.842	< 0.001[bc]
TS < 90% (%)	0.63(0.09,1.47)	0.2(0,1.17)	11.97(0.51,36.60)	H=23.066	< 0.001[bc]

Note: [a] P<0.05 between control and OB. [b]P < 0.05 between control and OB-OSAS. [c]P< 0.05 between OB and OB-severe OSAS.

ANOVA for F values; Kruskal–Wallis for H values ; Chi-square test for χ^2 values

Table 3. Comparison of cardiopulmonary exercise test indexes

Items	control(n=33)	OB(n=36)	OB- severe OSAS(n=45)	F or H	P
FVC(L)	3.59(3.13,4.31)	3.26(2.65,3.89)	3.73(3.10,4.47)	H=5.140	0.077
FVC%Pred(%)	94.70±16.00	83.42±15.47	83.56±15.11	F=6.139	0.003[ab]
FEV1%Pred(%)	88.00(83.00,99.00)	81.50(70.50,90.75)	85.00(75.00,91.50)	H=6.363	0.042[a]
FEV1/FVC(%)	83.00(79.00,90.50)	81.00(75.00,85.00)	80.00(74.50,87.00)	H=6.167	0.046
PEF(L/s)	6.04±2.02	5.15±1.79	6.05±2.14	F=2.473	0.089
PEF%Pred(%)	76.00(61.00,87.00)	68.50(50.00,77.75)	74.00(61.00,83.50)	H=4.215	0.121
MVV(L/min)	100.45（89.25,127.40）	98.88(78.40,112.00)	109.90(91.53,128.28)	H=4.293	0.117
VO_{2AT}(L/min)	1.08（0.83,1.28）	1.17（0.96,1.24）	1.04(0.88,1.25）	H=2.024	0.364
VO_{2AT}%Pred(%)	47.00(42.00,56.00)	48.50(41.00,56.75)	38.00(32.00,44.00)	H=20.035	<0.001[bc]
VO_{2AT}/kg(ml/min/kg)	14.90(13.05,18.20)	11.70(10.08,13.55)	10.70(9.15,12.25)	H=36.016	<0.001[ab]
VO_{2peak}(L/min)	2.10(1.77,2.60)	2.08(1.82,2.67)	2.42(1.90,2.84)	H=0.222	0.895
VO_{2peak}%Pred(%)	78.94±10.78	71.86±11.64	64.80±16.88	F=10.137	<0.001[abc]

VO_{2peak}/kg(ml/min/kg)	24.20(21.20,29.95)	17.20(15.70,20.05)	18.00(15.25,21.10)	H=43.168	<0.001 [ab]
HR_{max} (beat/min)	165.55±12.68	154.31±17.20	142.73±16.69	F=19.991	<0.001 [abc]
HR_{max}%Pred(%)	91.24±9.00	81.53± 9.69	79.56±7.74	F=18.369	<0.001 [ab]
RER_{max}	1.28(1.18,1.35)	1.25(1.20,1.33)	1.27(1.23,1.42)	H=3.106	0.212
HRr	55.97(44.27,67.33)	45.48(30.06,66.40)	41.56(29.29,62.12)	H=9.805	0.007[b]
VO_2/HR_{max} (ml/beat)	9.60(8.30,11.85)	10.40(9.43,14.05)	12.30(10.10,14.45)	H=9.971	0.007[b]
VO_2/HR_{max}%Pred(%)	79.00(68.50,89.50)	65.00(53.25,77.75)	59.00(48.50,69.50)	H=20.188	<0.001[ab]
BR(%)	50.79±14.91	43.61±16.72	47.60±17.91	F=1.615	0.204
WR_{max} (W)	126.00(113.5,159.00)	125.00(112.75,152.00)	146.00(117.00,175.00)	H=2.252	0.324
WR_{max}%Pred(%)	84.03±13.24	71.47±11.47	66.96±12.91	F=18.130	<0.001 [ab]
VE_{max}(L/min)	51.97(42.34,64.00)	51.98(42.97,65.39)	55.29(47.24,71.69)	H=3.245	0.197
VE_{max}%Pred(%)	76.39±14.14	57.97±17.47	55.38±16.20	F=18.196	<0.001[ab]
VE/VCO_2 slope	22.30(18.55,22.83)	22.02(19.78,24.00)	23.07(20.92,26.83)	H=5.067	0.079

Note: [a] $P<0.05$ between control and OB. [b]$P < 0.05$ between control and OB- severe OSAS. [c]$P< 0.05$ between OB and OB-severe OSAS.

ANOVA for F values; Kruskal–Wallis for H values ; Chi-square test for χ^2 values

Table 4. Intergroup comparison of cardiopulmonary exercise test indexes

		$FVC\%Pred(\%)$		$FEV1\%Pred(\%)$		$FEV1/FVC(\%)$		$VO_{2AT}\%Pred(\%)$	
Intergroup Comparison		mean difference	P	H	P	H	P	H	P
control	OB	11.280	0.003	19.580	0.042	16.794	0.104	1.525	1.000
	OB- severe OSAS	11.141	0.002	13.777	0.207	17.041	0.073	29.103	<0.001
OB	OB- severe OSAS	-0.138	0.968	-5.803	1.000	0.247	1.000	27.578	0.001
		$VO_{2AT}/kg(ml/min/kg)$		$VO_{2peak}\%Pred(\%)$		$VO_{2peak}/kg(ml/min/kg)$		HR_{max} (beat/min)	
Intergroup Comparison		H	P	mean difference	P	H	P	mean difference	P
control	OB	33.914	<0.001	7.078	0.035	46.207	<0.001	11.240	0.004
	OB- severe OSAS	43.814	<0.001	14.139	<0.001	43.640	<0.001	22.812	<0.001
OB	OB- severe OSAS	7.900	0.855	-7.061	0.024	-2.567	1.000	11.572	0.001
		$HR_{max}\%Pred(\%)$		HRr		VO_2/HR_{max} (ml/beat)		$VO_2/HR_{max}\%Pred(\%)$	

Intergroup Comparison		mean difference	P	H	P	H	P	H	P
control	OB	9.715	<0.001	16.711	0.108	-12.975	0.310	29.758	0.001
	OB- severe OSAS	11.687	<0.001	23.441	0.006	-23.897	0.005	41.024	<0.001
OB	OB- severe OSAS	1.972	0.316	6.731	1.000	-10.922	0.418	11.267	0.382
		WR_{max}%Pred(%)		VE_{max}%Pred(%)					
Intergroup Comparison		mean difference	P	mean difference	P				
control	OB	12.558	<0.001	18.422	<0.001				
	OB- severe OSAS	17.075	<0.001	21.016	<0.001				
OB	OB- severe OSAS	4.517	0.111	2.594	0.472				

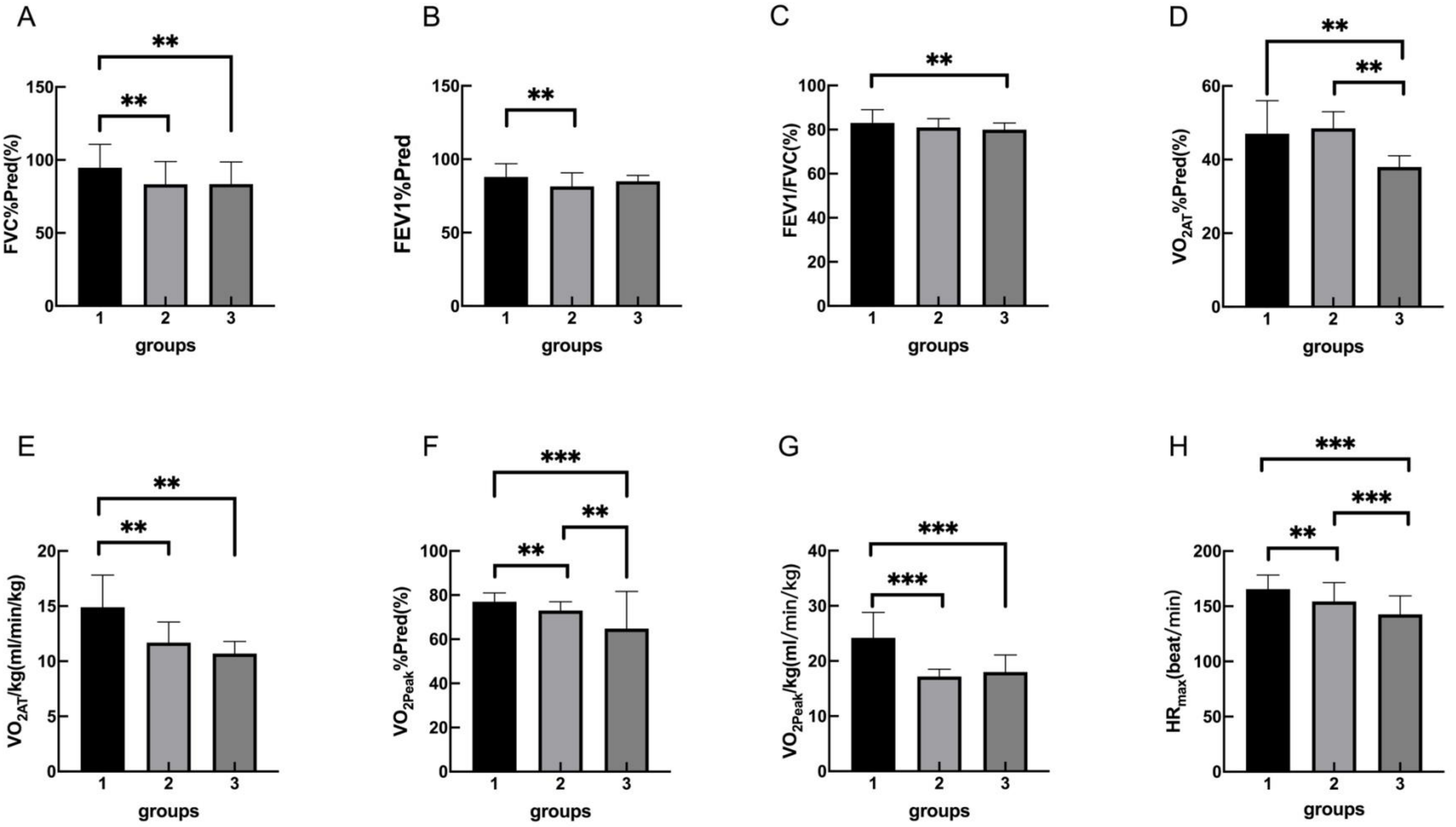

Figure 1

Group 1, 2, 3 represents the control group, OB group,OB-severe OSAS group.respectively. The comparison of cardiopulmonary exercise test indexes for three groups on FVC%Pred (A), FEV1%Pred (B), FEV1/FVC (C), VO_{2AT}%Pred (D), VO_{2AT}/kg (E), VO_{2peak}%Pred (F), VO_{2peak}/kg (G), HR_{max} (H). **$p < 0.05$, ***$p < 0.001$.

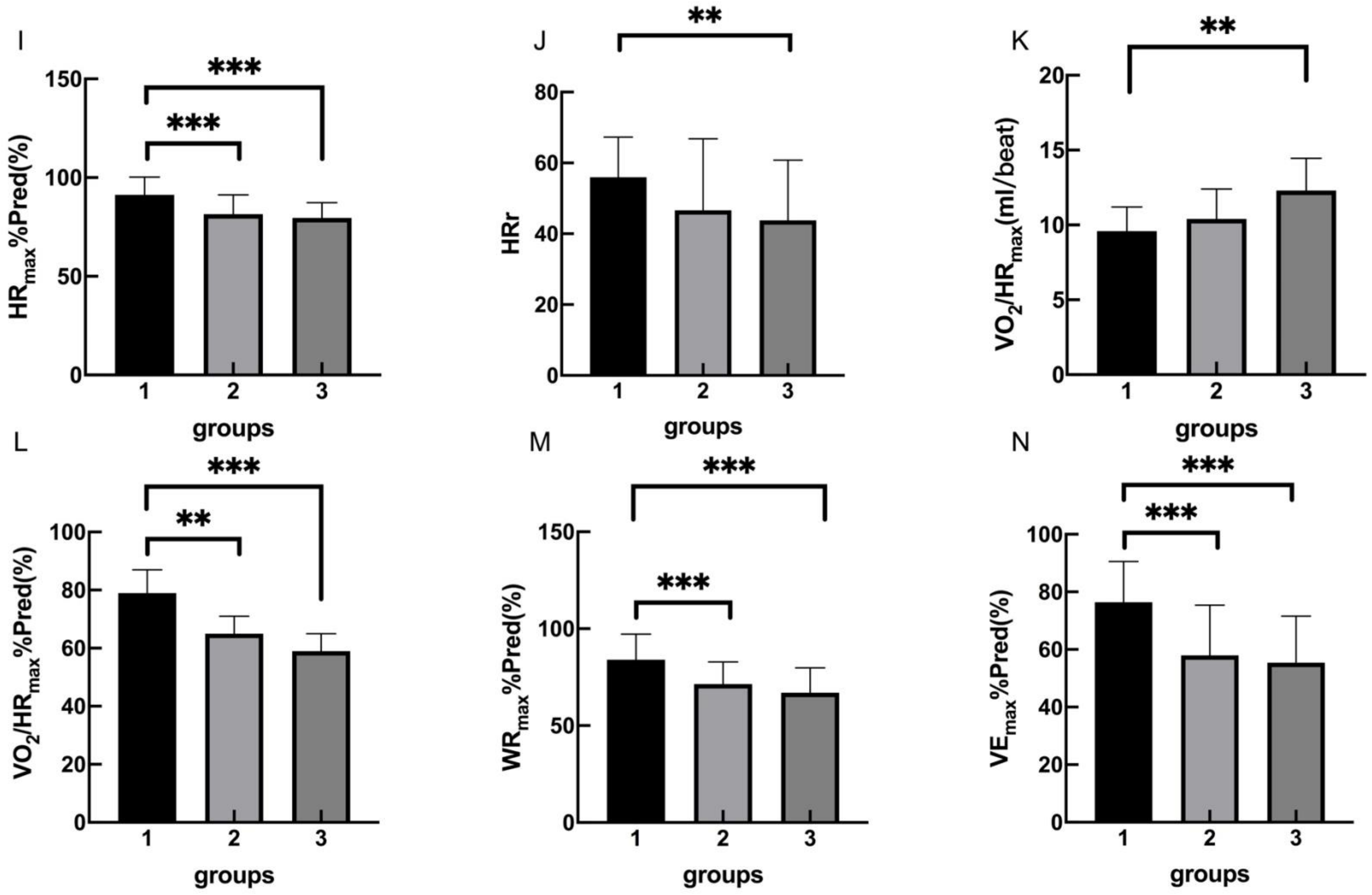

Figure2

Group 1, 2, 3 represents the control group, OB group,OB-severe OSAS group.respectively. The comparison of cardiopulmonary exercise test indexes for five groups on HR_{max}%Pred (I), HRr (J), VO_2/HR_{max} (K), VO_2/HR_{max}%Pred(L), WR_{max}%Pred(M), VE_{max}%Pred (N). **$p < 0.05$, ***$p < 0.001$.

Table 5.Correlation analysis between AHI and the observed indexes

Items	AHI	
	Correlation coefficient (r)	p values
Sleep efficiency (%)	-0.507**	< 0.001
$minSaO_2$ (%)	-0.737**	< 0.001
$meanSaO_2$ (%)	-0.651**	< 0.001
TS < 90% (%)	0.422**	< 0.001
VO_{2AT}(L/min)	-0.002	0.979
VO_{2AT}%Pred(%)	-0.351**	< 0.001
VO_{2AT}/kg(ml/min/kg)	-0.322**	< 0.001
VO_{2peak}(L/min)	-0.002	0.985
VO_{2peak}%Pred(%)	-0.349**	< 0.001
VO_{2peak}/kg(ml/min/kg)	-0.273**	0.003
HR_{max} (beat/min)	-0.434**	< 0.001

HR_{max}%Pred(%)	-0.340**	< 0.001
RER_{max}	-0.110	0.243
HRr	-0.200*	0.033
VO_2/HR_{max} (ml/beat)	0.247**	0.008
VO_2/HR_{max} Pred(%)	-0.354**	< 0.001
BR(%)	0.090	0.340
WR_{max} (W)	0.110	0.245
WR_{max}%Pred(%)	-0.377**	< 0.001
VE_{max}(L/min)	-0.077	0.416
VE_{max}%Pred(%)	-0.359**	< 0.001
VE/VCO2 slope	-0.104	0.273
FVC(L)	0.164	0.080
FVC%Pred(%)	-0.128	0.175
FEV1%Pred(%)	-0.035	0.709

FEV1/FVC(%)	-0.130	0.169
PEF(L/s)	0.103	0.274
PEF%Pred(%)	0.010	0.915
BMI(kg/m^2)	0.240*	0.010

Note: **p < 0.01,*p < 0.05

Table 6.Univariate and Multivariate Linear Regression Analyses of AHI Association with CPET Parameters

Items	Univariate Model					Multivariate Model				
	B	95%CI	R^2	t	p	B	95%CI	Adj.R^2	t	p
VO_{2AT}%Pred(%)	-5.301	(-8.146,-2.457)	0.109	-3.693	<0.001	-5.292	(-8,216,-2.367)	0.143	-3.587	0.001
VO_{2AT}/kg(ml/min/kg)	-1.681	(-2.678,-0.685)	0.091	-3.343	0.001	-1.661	(-2.715,-0.606)	0.074	-3.122	0.002
VO_{2peak}%Pred(%)	-6.063	(-9.264, -2.861)	0.112	-3.752	<0.001	-5.956	(-9.272, -2.639)	0.133	-3.560	0.001
HR_{max} (beat/min)	-9.970	(-13.720, -6.221)	0.199	-5.269	<0.001	-8.481	(-12.297, -4.664)	0.245	-4.406	<0.001
HR_{max}%Pred(%)	-4.209	(-6.364, -2.055)	0.118	-3.870	<0.001	-3.950	(-6.237, -1.662)	0.096	-3.423	0.001
VO_2/HR_{max}Pred(%)	-6.898	(-10.878, -2.917)	0.095	-3.433	0.001	-6.748	(-10.873, -2.623)	0.117	-3.423	0.002

WR_{max}%Pred(%)	-6.742	(-9.778, -3.705)	0.147	-4.399	<0.001	-6.121	(-9.222, -3.020)	0.191	-3.914	<0.001
VE_{max}%Pred(%)	-7.793	(-11.734, -3.852)	0.121	-3.918	<0.001	-7.307	(-11.435, -3.179)	0.123	-3.510	0.001
